# The use of legal empowerment to improve access to quality health services: a scoping review

**DOI:** 10.1186/s12939-022-01731-3

**Published:** 2022-09-16

**Authors:** Anuradha Joshi, Marta Schaaf, Dina Zayed

**Affiliations:** 1 Institute of Development Studies, Library Road, Brighton and Hove, East Sussex, BN1 9RE UK; 2 Independent Consultant, 357 Sixth Ave, Brooklyn, NY 11215 USA

**Keywords:** Legal empowerment, Human rights, Community development, Community health

## Abstract

This paper presents the results of a scoping review that examines the extent to which legal empowerment has been used as a strategy in efforts to improve access to quality health services in low- and middle-income countries. The review identifies lessons learned regarding legal empowerment program strategy, as well as impact on health empowerment and health outcomes, research gaps, areas of consensus and tension in the field.

The review included three main sources of data: 1) peer-reviewed literature, 2) grey literature, and 3) interviews with key legal empowerment stakeholders. Peer-reviewed and grey literature were identified via keyword searches, and interviewees were identified by searching an organizational database and snowball sampling.

The key findings were: first, there is very limited documentation on the use of legal empowerment strategies for improving health services. Second, the legal empowerment approach tends to be focussed on issues that communities themselves prioritize, often narrowly defined local challenges. However, legal empowerment as a strategy that pursues collective and individual remedies has the potential to contribute to structural change. Third, for this potential to be realised, legal empowerment entails building capacity of service providers and other duty bearers on health and related rights. Finally, the review also highlights the importance of trust—trust in state institutions, trust in the paralegals who support the process and trust in the channels of engagement with public authorities for grievance redress.

Several gaps also became evident through the review, including lack of work on private health providers, lack of discussion of the ‘empowerment’ effects of legal empowerment programs, and limited exploration of risk and sustainability. The paper concludes with a caution that practitioners need to start with the health challenges they are trying to address, and then assess whether legal empowerment is an appropriate approach, rather than seeing it as a silver bullet.

**Supplementary Information:**

The online version contains supplementary material available at 10.1186/s12939-022-01731-3.

## Introduction

Current global and national health efforts centre on the twin challenges of public service quality and inclusion, touting progress in both areas as fundamental to public health and human rights. This focus stems from the recognition that inequities and poor quality services undercut progress on public health indicators, often in a downward spiral, with discrimination and/or poor quality care causing people to avoid the health system when they can [1, 2]. As a general rule, the poor and otherwise marginalized are more likely to experience low-quality care than the population as a whole [3]. Thus, the Sustainable Development Goals seek to achieve Universal Health Coverage (UHC) of high-quality health services in a way that “leav[es] no one behind.” This aspiration is reflected in sector-specific strategies, such as the UN Secretary General’s Global Strategy for Women’s, Children’s, and Adolescents’ Health, which mainstreams quality, accessible and acceptable health services as germane to progress [4].

In addition to these government-led efforts to expand health care availability, accessibility, and quality, some community-based efforts aim to tackle the governance, rights, and technical challenges that underlie gaps in health care access and quality. This paper explores one of these approaches—legal empowerment—to understand how it may help to improve access to quality health care in low- and middle-income countries (LMICs). In the past decade, legal empowerment programs, using strategies such as community mobilization, legal literacy, community-based paralegals, and right to information laws, have been increasingly used to improve the access of marginalized and underserved populations to basic public services [5, 6]. At its core, legal empowerment aims to empower people to ‘know, use and shape the law’ to advance their rights to key public services [7]. In the health field in particular, where there is a substantial body of knowledge on the barriers to accessing quality healthcare, the legal empowerment approach is gaining currency as it addresses some key constraints, such as lack of rights knowledge among communities, lack of documentation required to access health services, discriminatory behaviour by providers, and capacity limitations in the health sector [8, 9]. Since 2008, when the United Nations convened a High-Level Commission on Legal Empowerment and the Poor to define key trends and questions in the field, legal empowerment programs have been tried in the health field in a wide range of settings, and for a wide range of issues (access to reproductive health care for women and girls; access to basic health care for indigenous groups and the poor; and securing rights of minorities including LGBTQ groups, persons with disabilities, stateless or identity-less groups among others [10, 11].

This paper is a scoping review that examines the extent to which legal empowerment has been used as a strategy in an effort to improve access to quality health services. Existing reviews of legal empowerment focus on the strategy overall, or on a sector other than health, such as property rights [12, 13]. Indeed, most existing reviews follow the conventions of law reviews, rather than social science evidence reviews [6, 12]. Uniquely, we consolidate existing evidence related to legal empowerment and health and frame this evidence in the context of theory related to governance, law reform and global health policy and priorities. The review identifies lessons learned regarding legal empowerment program strategy, as well as impact on health empowerment and health outcomes, research gaps,  and areas of consensus and tension in the field.

## Legal empowerment: conceptual clarifications

Legal empowerment approaches are consistent with evolving thinking in both the governance and public health communities about how to address persistent gaps in access and quality of health and justice services. As a defined area of practice, legal empowerment grew out of recognition that top down rule of law approaches on their own were limited in their ability to affect change in people’s every day engagements with the state, including their access to justice and to public services [14]. This recognition has corollaries in the broader development field, where problem-driven programming and vertically integrated accountability approaches are understood to be crucial complements to top-down programmes in order to address the multi-level determinants of accountability failures [15, 16]. In addition, much of the early conceptual framing of legal empowerment points to the limits of focusing on formal laws and policies, noting that informal practices are key in shaping individuals’ rights realisation, though these critiques were focused on practices related to property ownership rather than access to public services [10, 17]. Similarly, global health advocates and researchers call for attention to informality, such as the crucial role that health systems “software,” or “ideas, interests, values, norms, and relationships” play in shaping services ([18, 19], pg. 2). Attention to software reflects lessons learned from earlier efforts that prioritized reaching health coverage goals, at times without adequate attention to quality and patient preferences [20], effectively instrumentalizing the poor as numbers towards a goal, rather than as individuals endowed with health rights. In sum, most legal empowerment programmes focus on the interface of people and the state; this interface can be enormously consequential in people’s lives, but shaping it has proven resistant to prevailing strategies in governance, development, and public health.

Legal empowerment has its own potential pitfalls. Poor access to justice often tracks intersecting social hierarchies that influence individuals’ understanding of their ‘right to have rights’ [21], their ability to leave the home to claim rights and to effectively make their case, their treatment by the justice system, and their exposure to risk and violence for rights claiming. Marginalisation and minoritization and concomitant exclusion from health care are not simply technical challenges that can be addressed by health outreach; exclusion from the health system and/or mistreatment by the system is a way that social hierarchies are perpetuated [22]. In brief, neither the law nor the health system are necessarily mechanisms for empowerment.

Legal empowerment has been defined in variety of ways. The Commission on Legal Empowerment of the Poor described legal empowerment as: “a process of systemic change through which the poor and excluded become able to use the law, the legal system, and legal services to protect and advance their rights and interests as citizens and economic actors” [10], pg. 3). Legal empowerment has also been described as ‘‘the use of law and rights to help increase disadvantaged populations’ control over their lives” ([11], pg. 67), “bottom-up efforts to help marginalized people to learn about law and policy, and to use this knowledge to obtain concrete improvements in a relatively short period” [9], pg. 2),‘‘opening new avenues for advocacy and action, providing concrete mechanisms for redress for rights violations. Legal empowerment can also set precedence, ultimately strengthening the legal and policy framework” [23], pg. 3). The simplest definition is one where legal empowerment “enhances the ability of people to know, use and shape the law in order to protect their rights”, to place “the power of the law in the hands of ordinary people” ([7], pg. 3).

From the above, it is clear that there is a great range: from narrow definitions that only cover rights violations related to national laws and policies, to a broad definition that includes violations of rights that are enshrined in international human rights law; from attempts to ensure existing laws are implemented to attempts to transform the law; and from engaging directly with the judicial system, to engaging with bureaucratic processes that translate laws into people’s experience of their rights. Legal empowerment efforts may also engage with customary law structures, but this appears to rarely be the case for efforts related to the health sector.

Legal empowerment is closely allied to several other approaches to social justice including social accountability, social inclusion, human rights advocacy and strategic litigation [24, 25]. The closest, social accountability, has been widely used in the health sector, for example through the use of community scorecards or community monitoring [26, 27]. These strategies rely on people leading a collective effort to claim accountability for the provision of public goods that are existing state obligations, and tackling some of the structural constraints to service provision. Unlike legal empowerment however, it does not emphasise grievance redress for individual rights violations. Another related approach, social inclusion, like legal empowerment, focuses on the most marginalised groups by seeking to dismantle key drivers of exclusion, yet it cannot directly redress rights abuses [28]. In contrast to these social approaches, strategic litigation aims to leverage individual cases of rights violations to demonstrate patterns of state failure and generate changes in law and policy through precedent setting. However, unlike legal empowerment, strategic litigation, on its own, is not rooted in the empowerment of affected people [29].

Some legal empowerment theorists also call for “critical legal empowerment,” which recognises the limits of the law as a tool for emancipation [30]. As a theory and an emerging approach in practice, critical legal empowerment integrates principles and strategies from successful social accountability, social inclusion, and strategic litigation efforts – namely collective action on the drivers of exclusion. Critical legal empowerment centres the individuals and communities who experience rights violations and focuses on the power dynamics that give rise to their marginalization [30]. Developing countervailing power among communities is essential to addressing structures of exclusion. In this framing, the law may be a tool or an impediment for emancipation, and as such, legal empowerment practitioners may seek to disseminate, use and shape the law as part of a collective struggle to change their political, economic, and social context. Focus on the law in the absence of such power analysis is insufficient, as it fails to recognise the ways that the law may reflect and perpetuate the status quo [30]. The critical legal empowerment approach offers several advantages in the context of health, where ambiguity regarding entitlements, laws that are at odds with public health evidence (particularly in the domain of sexual and reproductive health and rights), and embedded social hierarchy relating to gender, race, caste and other issues both shape health status and undermine the use of law as a tool for emancipation [30].

**Fig. 1:**
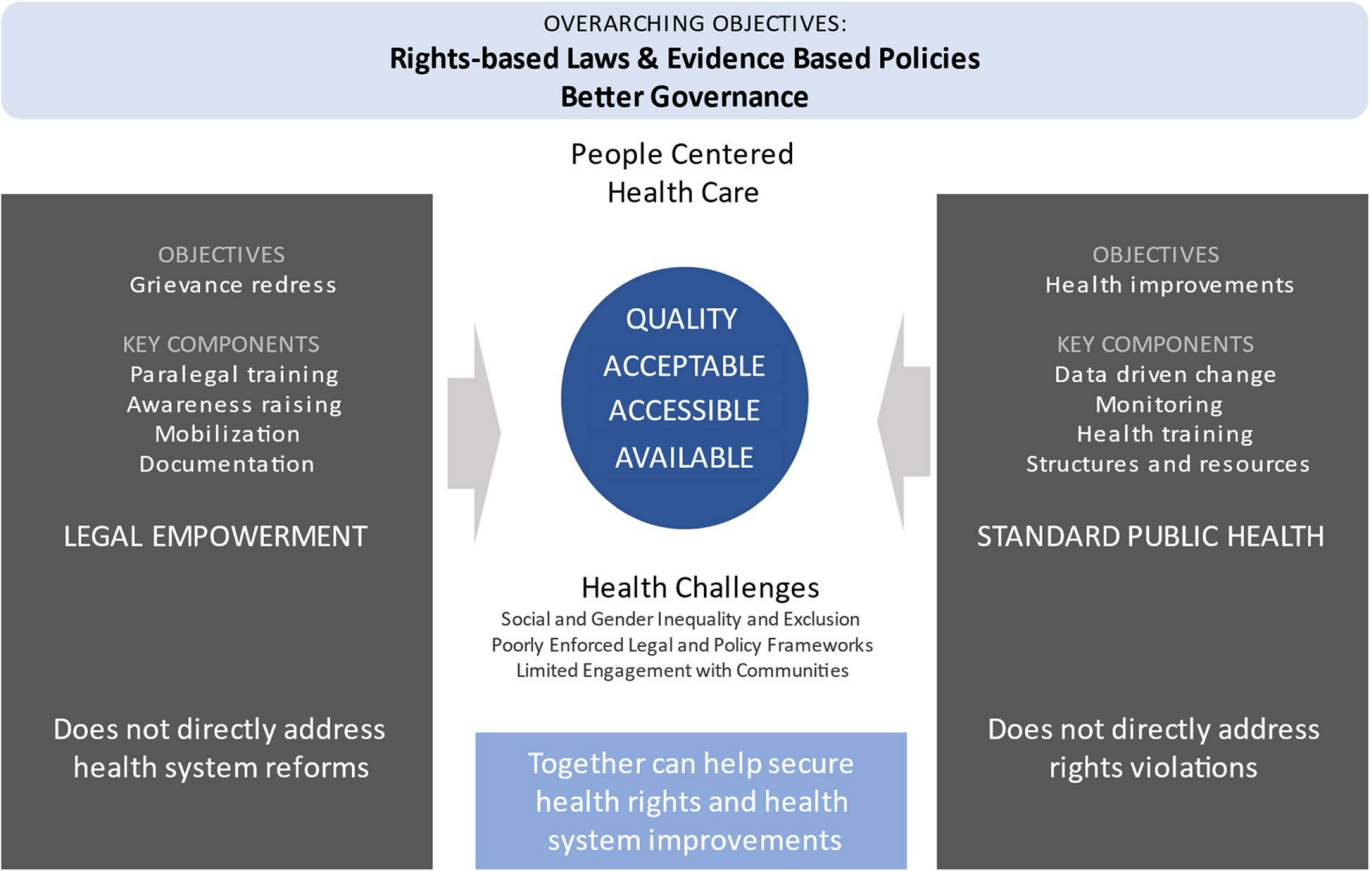
Complementarity between legal empowerment and standard health approaches

In order to clarify the potential benefit that legal empowerment can bring to the delivery of health services, we present a framework in Fig. 1 for thinking about how each contributes to improving access to quality health services. The figure highlights the challenges that are embedded in health systems such as social and gender inequalities, poorly enforced health policy, and limited engagement with communities; as well as social structures and norms that impede people from seeking and/or receiving the high-quality health services to which they are entitled.

Conventional approaches to improving health service availability, accessibility, acceptability and quality tackle challenges through top-down strategies and programmes that seek to improve care utilisation and effectiveness. Such typical approaches rely on data and monitoring to inform and assess activities such as behaviour change among communities to spur health care use, training for health workers, the creation of new monitoring and improvement processes, and the provision of additional resources.

In addition to conventional public health approaches to tackling these challenges, legal empowerment seeks to make people active agents in ensuring their own access to services. The key elements of the legal empowerment approach are awareness raising of health rights; collective mobilization to tackle shortcomings; documentation of rights violations; and training, deployment, and support of paralegals to help individuals to navigate the grievance redress process.

The expectation is that these approaches working together will lead to responsive and fair administrative and legal processes, improvements in the grievance redress process, and usable channels for policy advocacy on the one hand; and a better trained health force, equitable resource allocation, responsive health systems, and improved citizen driven accountability on the other hand. Ultimately the functional grievance redress system and improved health systems governance can ensure that health services are available, accessible, acceptable and of good quality.

By placing the two fields together in one diagram, one can see the gap in addressing rights violations that taking a legal empowerment approach to health services can fill. Legal empowerment can provide a tool for people to directly address failures of health systems to provide them accessible quality services, through grievance redress systems including litigation, by collaborating with health and other professionals to improve systems, and through advocacy aimed at law and policy makers to change the law. Thus, legal empowerment offers an additional pathway to shifting the power in health systems, from government functionaries to the people who are supposed to be at the heart of health services, ultimately shaping the interface of the patient and the state.

## Methods

This review aimed to build on the conceptual framework elaborated above by assessing the empirical evidence regarding legal empowerment and health.

Our research questions were: What is the state of evidence regarding legal empowerment and health in low- and middle-income countries (LMICs)? Specifically, what are the outcomes documented, what are the challenges, what are the barriers, and what gaps and learning priorities emerge?

The review was focused on LMICs.^1^

For the purposes of this review, we define legal empowerment as efforts to:

- help marginalised people to become aware of domestic laws and policies that confer entitlements,
- facilitate collective mobilisation to improve law and policy and/or its implementation, and,
- use these laws and policies to address grievances related to access to quality health services, by engaging with relevant administrative and legal systems.

We used the United Nations Economic and Social Committee’s General Comment 14 on the Right to the Highest Attainable Standard of Health to frame analysis of how legal empowerment affected the right to health. General Comment 14 explains that health facilities, goods and services, and programmes should be Available, Accessible, Acceptable, and of good Quality (AAAQ).

The review included three main sources of data: 1) peer-reviewed literature, 2) grey literature, and 3) interviews with key legal empowerment stakeholders. We included grey literature and interviews because, based on our prior knowledge of the field, we knew that the peer-reviewed literature was thin. We chose a scoping review approach because our research questions were broad, seeking to map the evidence and assess gaps in a heterogenous literature [31]. We followed the standard scoping review steps of identifying the research question, identifying relevant studies; study selection; charting the data; and collating, summarizing, and reporting the results [32].

For both the peer reviewed and grey literature we identified two types of papers: papers that described programme experiences at the intersection of legal empowerment and access to quality health services (“empirical papers”), and more general papers that were relevant to this focus but did not describe a specific programme in detail (“background papers”). We describe the methodology for each of these in turn.

### Peer-reviewed literature

To ensure that we included a wide variety of literature we searched PubMed, Nexis Uni, GoogleScholar, ProQuest Dissertation, and EThOS. Search terms included: (legal empowerment, OR paralegal, OR patient navigat*), AND health. Based on a title and abstract screen and excluding duplicates, we imported a total of 297 peer-reviewed papers to the folder of “empirical” resources in Zotero for full review.

The inclusion criteria applied to the peer reviewed empirical resources are presented in Table 1. The three authors (AJ, MS, and DZ) jointly assessed the full text of ten of the 297 papers for inclusion/exclusion to ensure that we were applying the criteria consistently. We then divided the rest of the papers among the three authors for full review, ultimately retaining 14 papers. Of the 297, we moved pertinent commentaries and conceptual papers that did not describe programmes from the “empirical” folder to a “background” folder in Zotero. Because of the heterogeneity of the literature reviewed and because our intent was scoping rather than meta-analysis, we did not apply any quality filters to the papers.

**Table 1:** Inclusion and exclusion criteria for the empirical paper review

Inclusion	Exclusion
The paper describes a legal empowerment programme addressing – but not necessarily exclusively—proximate determinants of health, such as health service access or quality, or social determinants of health that have an immediate impact on health and that are framed as a health determinant in the paper, such as water/sanitation	The paper describes a legal empowerment programme addressing distal determinants of health (e.g. interaction with the criminal justice system or housing), and is not framed in terms of health
As per our definition of legal empowerment, the paper describes a programme that starts from entitlements that are formally enshrined in domestic law or widely understood in customary law, has an element of grievance redress for individuals, is part of a collective effort to mobilize people to raise awareness and challenge violations, and is embedded in communities	Irrespective of whether the paper refers to the programme described as “legal empowerment,” the programme described does not meet the definition of legal empowerment we are applying in this review
Paper describes a legal empowerment programme addressing health in a low- or middle-income country	Paper describes a legal empowerment programme addressing health in a high-income country
Paper is in the English, Arabic, or French languages	Paper is in a language other than English, French, or Arabic

We then conducted similar searches in French and Arabic.^2^ The French and Arabic search terms were selected based on a review of how the English terms were translated in UN and other international organisation documents, and, in the case of Arabic, we added additional search terms suggested by practitioners in the Arab-speaking Middle East and North Africa, reflecting the fact that the use of translation and synonyms can fail to capture key accountability constructs and principles [33]. Following title and abstract review of the search results, we did not add any French or Arabic language papers to Zotero for full review.

### Grey literature search

We conducted the same English, French, and Arabic searches as we used in GoogleScholar in Google, limiting our results to PDFs. We stopped reading the search results after 150 records in a row were not relevant. As a result, we added 2 English language records into the “empirical” folder in Zotero. The French papers identified were translations of reports we already had in English. For the Arabic search, the broader Google search yielded an additional 15 resources. None of the 15 papers found met the requirements for our empirical folder; they were all added to the background folder in Zotero.

We also asked interviewees for suggested resources, yielding additional papers for the “background” folder.

### Interview strategy

Key informant interviews provided complementary information on our emerging findings, and helped us to identify additional grey literature materials to be included in our review. Though we did not undertake a formal consultation, the interviews served a similar purpose to the optional consultation stage of scoping reviews described by Arskey and O’Malley and Levac et al. [32].

Interviewees were recruited using an introductory email explaining the purpose of the review. All three authors conducted interviews using a semi-structured interview guide that helped us to explore the field relevance of our conceptual framework and probe emergent findings from the peer reviewed and grey literature reviews. Because the objective of the interviews was to confirm and explore emerging findings and identify new grey literature materials, it was not necessary to record or transcribe the interviews. The interviews added detail and rigor to our analysis, and did not aim to explore the lived experience of the interviewee or other more complex phenomena typical of qualitative research and analysis. Thus, the interviewers took detailed notes.

We identified the 21 individuals interviewed by starting with people we already knew, searching an international NGO (Namati’s) legal empowerment community of practice database, and snowball sampling. The individuals interviewed included legal empowerment and health donors (*n* = 2), researchers (*n* = 3), global experts (*n* = 5) and practitioners (*n* = 11).

### Analysis methodology

We recorded key details of the peer-reviewed (*n* = 14) and the grey (*n* = 2) literature empirical articles in an extraction tool, which included fields for the programme description, approach to legal empowerment, legal/policy basis for claims, target population, outcomes, facilitators, and key challenges, among other areas. We then synthesised these fields into brief memos, comprising the basis of our findings.

We created a limited coding system for the 121 background papers. The codes we used were instrumental and related to the research questions, including, for example, enabling factors, barriers, knowledge gaps, and research priorities. Codes were then distilled into brief summaries; we subsequently coded interviewers and integrated findings from the interviews into these summaries.

## Results

We first describe the parameters of the empirical literature on legal empowerment and health, and then summarise the impacts documented on AAAQ of health services and determinants, the strategic and programmatic approaches taken to legal empowerment and health, as well as enabling and constraining factors.

### Parameters of the empirical literature on legal empowerment

This section summarises the parameters of the empirical articles identified in the peer-reviewed (*n* = 14) and grey (*n* = 2) literature. As outlined in Supplemental Table 1, the 16 empirical articles described 22 programmes that met our definition of legal empowerment. We tried to avoid double counting programmes that are represented more than once in the literature, but these distinctions were not always clear. We had to make judgment calls as articles described the programme at different points in time, described different elements of the same programme, or presented activities implemented by different NGOs as one programme, as they were part of a comprehensive strategy funded by one donor. All of these programmes were implemented by NGOs. Of the programmes we reviewed, the majority targeted ‘the poor’ as un undifferentiated category, sometimes in a given locale, such as an urban slum (*n* = 5) [8, 9, 24, 34, 35]; people living with HIV or described as a “key population” for HIV concern, namely people who use drugs or commercial sex workers (*n* = 8) [36, 37, 38, 39, 40, 41], women, including women survivors of gender-based violence (*n* = 3) [35, 42, 43] and indigenous populations and the Roma (*n* = 5) [24, 44, 45]. Women are often noted as a priority population within the main population addressed, *e.g.* extra efforts to reach women who use drugs. All of the papers described programs seeking to improve the availability, accessibility, acceptability, and/or quality of public sector health services.

Most of the programmes reviewed employed paralegals as a centrepiece of their legal empowerment strategy. The stated aims of most legal empowerment work were dual: a) the improvement of the AAAQ of health services and better fulfilment of health entitlements and b) the empowerment of the focus populations, although different organisations had varying conceptions of what empowerment meant; some did not describe what empowerment meant at all. An additional objective for the work of a small subset of organisations was improvements in what they framed as proximal determinants of health, namely the reduction of arrests and/or violence against certain populations (usually sex workers and drug users or women), and improved respect for the property rights of recently widowed women [36, 37, 40, 41].

Overall, there was little explicit articulation of the theory of change underpinning the programmes described, although there were implicit assumptions being made, for example that providing individuals with information about their rights and entitlements would spur them to claim those rights. Some programmes seemed to work adaptively rather than with an explicit theory of change, through trial and error, abandoning avenues that bore little fruit: e.g. in Guatemala the Center for the Study of Equity and Governance in Health Systems (CEGGS in Spanish) abandoned the approach of quantitatively documenting and summarising cases in favour of narrative storytelling as storytelling seemed to have more traction with both politicians and public health officials [24].

Of the 16 empirical papers, few were formal evaluations of legal empowerment and health programmes. Exceptions include Abdikeeva and Covaci [44], which carried out a qualitative baseline and end-line assessment around four strategies, legal empowerment, documentation and advocacy, media advocacy and strategic litigation; Schaaf and colleagues [9], which carried out retrospective case reviews of grievances and their resolution; and Gruskin and colleagues [40], which included a resource assessment questionnaire, a review of programme records and routine data, and semi-structured interviews and focus group discussions with clients and service providers. The remainder of the papers might best be described as programme summaries, commentary and reflection; largely qualitative and limited in scope, drawing on focus groups, interviews, and programme information. Because the programme assessments are limited, the observed outcomes are anecdotal and lack clear attribution or contribution analysis. Understandably, these programme summaries and studies cannot link programme activities to health outcomes or to measurable changes in health service availability, accessibility, acceptability, or quality.

### Impacts of legal empowerment programme activities on the AAAQ of health services

In Table 2, we describe how each programme reviewed in the empirical literature addressed and/or impacted the availability, accessibility, acceptability, and quality of health care services and proximal health determinants. We did not include interviews in this table, as we did not always get full details on the programme impacts from interviewees. Moreover, in order to make key conclusions about the state of research on legal empowerment and health reproducible, we kept this portion of our analysis confined to the published literature. We report on both activities and impacts, consistent with the ways the papers themselves framed their summaries. Moreover, there are some activities that could be described as improving two elements of the AAAQ framework, such as acceptability and quality. We chose the element that we thought corresponded best to the framing in the paper. Many of the organisations profiled in the papers engage in a range of activities, such as media advocacy and strategic litigation. We only include the AAAQ impact of the legal empowerment activities here.

**Table 2:** Impact of legal empowerment programs on healthcare and proximate health determinants

Authors	Country	Availability	Accessibility	Acceptability	Quality	Action on health determinants
Wirya, A; Larasati, A; Gruskin, S; Ferguson, L	Indonesia	Paralegals ensure that their clients obtain health services by taking medicines directly to them	Paralegals help clients to be more aware of their health-related rights, especially regarding their rights to obtain health services inside detention and encourage law enforcement agencies to refer the clients to health services			Paralegals try to reduce incidence of violence against clients and others
Dhital, S & Walton, T	Pertinent parts of the article focus on India	In response to community request, the implementing NGO secured emergency relief kits during the first COVID lockdown	The implementing NGO provided training to paralegals and community organisers regarding health and social protection entitlements specific to COVID-19. During the pandemic, the NGO also filed complaints and petitions when community members were unable to access COVID-related and other health entitlements			
Gruskin S.; Safreed-Harmon, K.; Ezer, T.; Gathumbi, A.; Cohen, J.; Kameri-Mbote, P	Kenya		Clients interviewed showed an increase in practical knowledge and awareness about how to improve their access to healthcare. Some were referred to mental health services	Clients interviewed showed enhanced ability to communicate with healthcare providers		
Abdikeeva, A. & Covaci, A	North Macedonia		Paralegals and others working for NGOs challenged violations such as the outright denial of reproductive health care and of drug dependence treatment, as well as health provider demands for bribes or other illicit payments. One NGO supported individuals to obtain papers required to access health care		The paralegals and others employed by the NGOs described challenged discriminatory treatment in healthcare settings	
Abdikeeva, A.; Ezer, T.; Covaci, A	Pertinent parts of the article focus on Romania and Serbia		NGO staff provide individual and community training on rights and entitlements	In Romania, they bring cases of discrimination and mistreatment to the College of Physicians	In Serbia, NGO staff train duty bearers on their responsibilities under national and human rights law to Roma patients	
Achilihu, I	Sierra Leone	Paralegals address availability challenges, such as inadequate staff for service delivery, and lack of vaccines and essential drugs at the clinic	This program provides legal education regarding health rights and entitlements to paralegals, Facility Management Committees, and community members. Paralegals address accessibility related problems such as demands that patients make informal payments			
Dworkin, S.; Lub, T.; Grabec, S.; Kwenad, Z.; Mwaura-Muirue, E.; Bukusid, E	Kenya		The implementing NGO provided supportive services to protect marginalised communities from discrimination and exploitation			This intervention trained paralegals, CHWs and others to assist recently widowed women to use the customary or formal legal system to protect their land rights to reduce their vulnerability to HIV infection
Biradavolu, M.R.; Burris, S.; George, A.; Jena, A.; Blankenship, K	India		NGOs and CBOs trained CSWs in their rights and entitlements to reduce abuse by the police, such as arbitrary arrest, improving their access to ongoing HIV prevention programmes			NGOs and CBOs engaged in collective action in order to decrease violence and abuse by the police towards CSWs
Kolisetty., A	Bangladesh		A paralegal (shebika) connects community members with legal advice, representation, and mediation services for a variety of issues including health			Paralegals use the customary and formal legal systems, as well as alternative dispute resolution to address a variety of direct determinants of health, including gender based violence and child marriage
Network Movement for Democracy and Human Rights	Sierra Leone		Paralegals undertake awareness raising on rights and entitlements among community members, and manage "cases" that arise from the monitoring, including as related to informal payments			
Feinglass, E.; Gomes, N.; Maru, V	Mozambique	The NGO trains paralegals (Health Advocates) and Village Health Committees raise community awareness about health rights and entitlements. Paralegals facilitate dialogue among communities and clinics and use formal administrative channels as well as dispute resolution skills to address particular gaps related to availability, such as lack of essential medicines	The paralegals also address access problems, such as demands for informal payments		The paralegals address quality challenges, such as rude treatment by providers	
Wolfe, D.; Cohen, J.; Doyle, H.; Margolin, T	Kenya, Indonesia, Ukraine		Through legal training, joint CSW/police workshops, and use of paralegals, the implementing NGOs sought to reduce police abuse that disrupted access to HIV prevention services			Through legal training, joint CSW/police workshops, and use of paralegals, the implementing NGOs addressed police violence against CSWs and others at risk for HIV
Jagannath, M.; Phillips, N.; Shah, J	Haiti				NGO staff and grassroots actors support women who have experienced rape and other types of GBV to receive better medical care and forensic examination	NGO staff and other grassroots actors also support women reporting rape and other GBV to demand and receive better treatment from police
Feruglio, F	Multiple (but for inclusion—only Kenya)		The implementing NGO provides health and legal awareness raising to female commercial sex workers and other women who are vulnerable to HIV, as well as to health providers and police officers. In addition, paralegals accompany women to trusted providers and facilities			The paralegals hope to reduce police abuse of CSWs and other vulnerable women
Schaaf, M; Falcao, J., Feinglass, E., Kitchell, E., Gomes, N., Freedman, L	Mozambique	Trained paralegals and Village Health Committees took a variety of steps to resolve cases regarding availability of key inputs, such as essential medicines. They educated health workers and administrators about how to solve a certain problem and assisted them to do it; facilitated a dialogue between the client and the allegedly offending provider; and helped the client and/or the health facility to use formal administrative processes to solve the problem	The paralegals also address access problems, such as demands for informal payments		The paralegals addressed key quality problems, such as women being forced to deliver alone and rude treatment	
Joshi, A	3 relevant: North Macedonia, Uganda, Guatemala	Paralegals in Guatemala (Community Defenders of the Right to Health) use formal redress mechanisms at local and national level to address basic availability issues, primarily lack of ambulance response and lack of essential medicines. Paralegals and NGO staff in Uganda (Community Health Advocates) mediation and judicial processes to address availability challenges, such as denial of care	Paralegals in Guatemala (Community Defenders of the Right to Health) address basic availability issues, primarily demands for informal payments. In North Macedonia, paralegals assist members of the Roma community in accessing legal identity documents required to access healthcare		Paralegals in Guatemala and Uganda address some quality issues, such as rude and/or discriminatory treatment. In North Macedonia, they address discriminatory and rude treatment	

As can be seen from the Table, most of the activities relate to access and quality, though availability and acceptability are addressed. In addition to improving health services, some programmes also seek to reduce exposure to ill health, such as dangerous environments or police violence. As noted, none of the articles provide evidence of impacts on health status, beyond anecdotal examples or speculation.

### Strategic and programmatic approaches taken to legal empowerment and health

This section describes the range of strategies and programmes which different actors employ to promote legal empowerment for health. Our review of the published evidence and the interview transcripts suggest that organisations try to address three common problems in accessing justice as related to health: i) lack of awareness among people regarding their rights to access quality health services, and also regarding possible grievance redress options ii) a scarcity of lawyers and access to legal systems to provide remedy in cases where health rights are denied, and iii) the insufficiency of juridical processes alone to address the determinants of state failure to realise health obligations enshrined in national law and policy. These are summarised in Table 3.

**Table 3:** Common health service issues addressed by legal empowerment

Problems addressed by LE programmes	Modalities	Examples
Community lack of awareness on health rights, entitlements, and tools for grievance redress	• Training • Awareness raising, such as “legal literacy classes” • Community scorecard process to document reality against standards • Creation of tools for low literacy populations • ‘Conscientization’ in Freirean tradition	• Train detained people on their rights regarding health care access in detention • Legal Counsellors from partner NGOs undertake assessments in communities of sex workers, people who use drugs, and others regarding their knowledge and priorities, and then conduct a training on entitlements and remedy • Community-based awareness raising sessions regarding the link between civil registration and health insurance • Creation of “Health Advising Centers” that conduct information sessions • Supporting collective efforts to gain health insurance
Poor access to systems to provide remedy and redress	• Community paralegal programmes (also called “Barefoot lawyers,” “Legal Counsellors”) • Mobile legal clinics • “Legal integration” programmes, where legal services are provided in health settings • Health Advising Centers that provide information and support to individuals • Training and collaboration with government, community-based structures, such as Village Health Committees or Health Facility Committees	• Paralegals inform providers about patient rights and entitlements and health sector policies or meet with people whose health needs are to be met (e.g. individuals in detention or women who require support with unwanted pregnancies) • Paralegals confront health providers and/or institute formal or informal complaints regarding denial of care, rude treatment, requests for bribes, or other types of mistreatments • Paralegals pressure/support providers and managers to address health system challenges, such as stock outs or absenteeism • Paralegals advise and accompany survivors of sexual violence on legal processes • An extensive network of outreach workers facilitates community member contact with paralegals and legal aid clinics • Referral to legal aid, pro-bono services, other complementary services • Registration support to establish legal personhood
Inability of judicial processes to address gaps in effective health coverage	• Documentation for advocacy	• LE programme aggregates cases to illustrate patterns of state failure related to effective health coverage gaps • LE programme maps violations/cases in order to illustrate troubled facilities

Several elements are common to many legal empowerment and health programmes. First, a common core of legal empowerment that is used to different degrees by various initiatives is community rights awareness raising and mobilisation regarding rights and entitlements that are enshrined in law and policy. This is often accomplished through training, which may be preceded by needs assessments of specific marginalised communities with the help of legal counsellors [41] or community scorecard like processes [46] to understand the context within which training is required (e.g. are the key issues for specific communities identity documents and civil registration, or health care access challenges or health entitlements related to detention, or others). In some cases, awareness raising relates not only to entitlements, but also aims to develop awareness of “legal and administrative provisions that place disproportionate burdens” on the communities in question, as the case of Roma in North Macedonia [44], pg. 104). A few programmes also include training in customary law as an avenue for establishing and claiming rights and remedy in addition to the formal legal system, as was done by Namati in Sierra Leone [34]. Also, in order to support the spread of rights awareness and rights claiming, community mobilisation activities sometimes engage existing community-based structures, such as Village Health Committees [8], Facility Management Committees [34] and grassroots associations [43].

Another core element that builds on the community awareness and mobilisation involves training community members as paralegals on rights and entitlements related to health as well as on the judicial, administrative, or customary processes that can be used to claim rights or provide remedies. These paralegals range from lay people who express an interest [8, 24] to individuals who are selected based on a role they already play, such as Community Health Workers (CHWs) [37]. In addition to their didactic training, such paralegals simultaneously develop valuable networks among actors such as health providers, administrators, legal professionals, law enforcement and others that they can turn to when seeking to resolve individual issues. Paralegals hone judgements on whether cases can be resolved informally through negotiation and collaboration, or alternatively, whether they need to invoke formal legal or administrative processes. In a couple of programmes we found, community paralegals and the police/health officials were offered training together, increasing mutual understanding and also developing networks that enabled paralegals to access officials directly, preventing case escalation [39, 47]. While individuals explicitly identified as paralegals are the linchpin of most legal empowerment and health programmes we identified, a few others relied on community-based volunteers who are not described as paralegals, on cooperation with local associations, or on other approaches.

Finally, most legal empowerment and health programmes include a documentation element. Documentation of rights violations helps in preparing and tracking cases of individual rights violations for programme monitoring and evaluation as it can be used as an internal monitoring tool that facilitates internal learning about types of cases, rates of resolution, and effective strategies [9]. Moreover, documentation also helps to develop an evidence base for broader advocacy for improvement of laws, policies and systems, as legal empowerment programme managers identify patterns in the data. Community paralegals or other programme staff are generally trained to document, monitor and file complaints in keeping with what the law requires if the complaint is to go to court [42]. Legal empowerment actors may also document the context more broadly, identifying challenges in health service access or other trends that can inform programme implementation and advocacy. CEGGS in Guatemala produces maps of documented cases, so one is able to identify facilities which are the site of multiple violations [24]. Similarly, interviewees indicated that paralegals working with Namati Mozambique and Nazdeek in India collate similar cases to advocate vis-à-vis sub-national or national structures to issue new directives, institute training, address infrastructure gaps, or otherwise take steps to address frequently occurring problems that undercut the AAAQ of health. A few interviewees noted that documentation was key to their advocacy vis-à-vis government actors, as it served as “evidence” regarding the current situation that also shed light on challenges that governmental officials may have been unaware of.

**Fig. 2:**
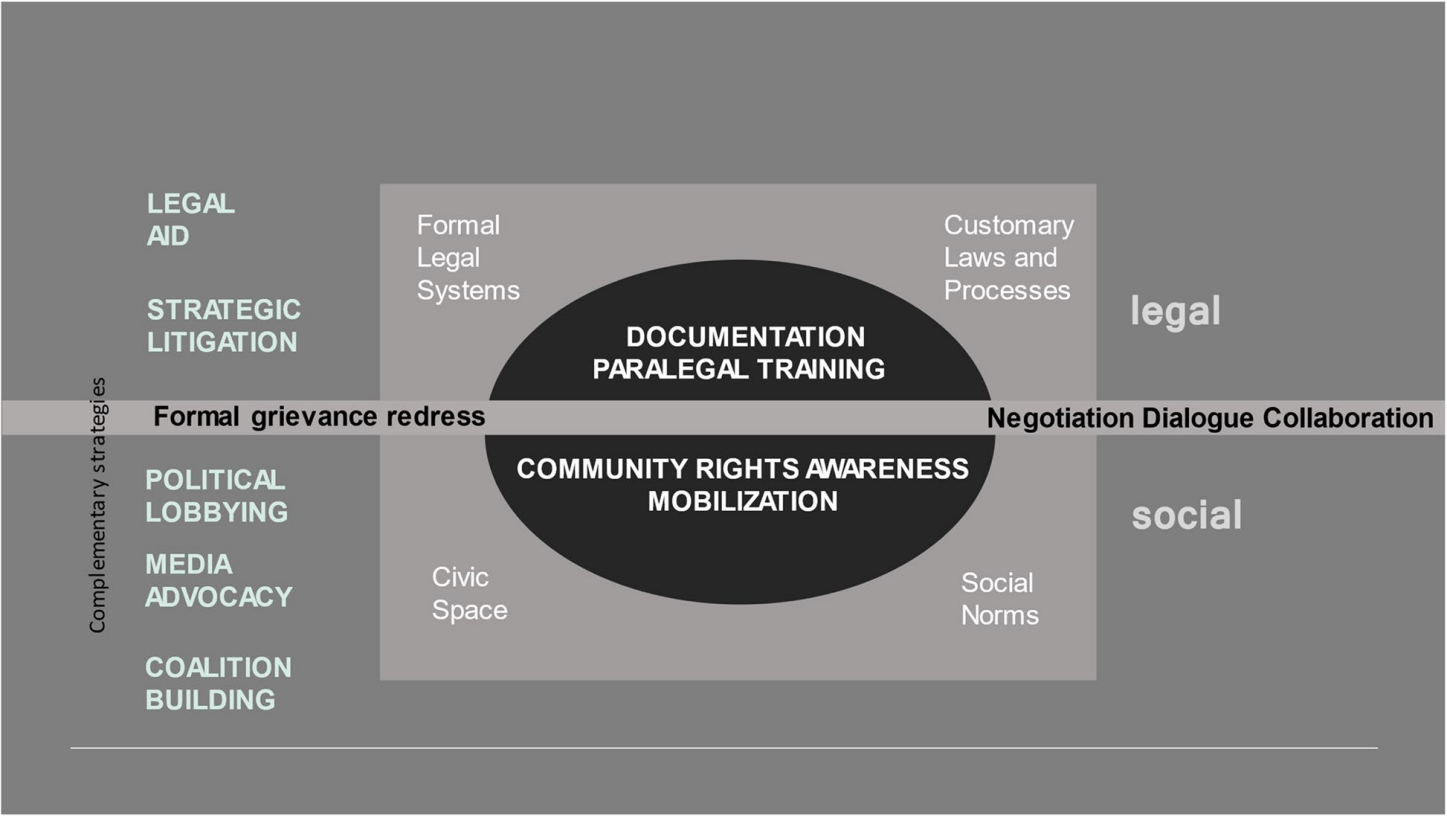
Legal empowerment within an ecosystem of social justice strategies

As depicted in the Fig. 2, in addition to the core elements described above, training of paralegals and communities on their rights is often accompanied by other approaches—both legal and political that are used in parallel: legal (strategic litigation, legal integration, legal aid, documentation of rights abuses) and political (media advocacy, community mobilization, community led research, budget advocacy, and political participation).

More specifically, a range of legal strategies may complement legal empowerment. Strategic litigation is used by several organisations to shift bureaucratic responses to rights violations by leveraging a single case to effect change at the level of law, policy, practice or social discourse [44]. Other programmes offer legal aid in addition to legal empowerment so that grievances can be pursued through the judicial system [47]. Several of these strategies can be used in tandem to build on each other. For example, NGO programmes addressing the health and other needs of people with HIV, survivors of gender-based violence, and other populations in Kenya use a legal integration strategy, which they define as, “programmes incorporating legal aid, training and representation, into existing health services to improve health outcomes, and advance human rights,” ([40], pg. 1).

In addition, many legal empowerment programme implementers employ political strategies to supplement the legal ones. Working with media, highlighting cases of rights violations, and bringing them to the attention of the broader public is a common strategy we saw in empirical articles as well as heard about in interviews [45]. Building networks and coalitions is another common approach. For example, in India, an NGO formed a Crisis Intervention Team that included politicians, government officials, and members of the media in addition to lawyers and human rights activists, in order to respond to arbitrary arrests of sex workers [36].

Most programmes combine legal and political approaches. For example, a programme implemented by a Haitian public interest law firm, Bureau des Avocats Internationaux (BAI) uses a combination of an explicitly political approach with a legal empowerment approach:

“BAI lawyers incorporate organizing strategies into their legal cases by using “grassroots coordinators” to reinforce the capacity of grassroots groups that work on the same issues for which the BAI’s clients seek legal assistance. The grassroots coordinators work with the BAI’s lawyers to organize rights trainings for grassroots groups and facilitate their advocacy efforts, such as engagement with the media, demonstrations, and meeting with government officials. This “victim-centered” approach deploys legal and political strategies that reinforce one another; the lawsuits provide a foundation for the communities’ organizing efforts while the political pressure helps advance cases through the courts and compel a judicial response” [43], pg. 10).

Some programmes reviewed used innovative angles to advance health rights, particularly those regarding key proximate determinants of health. For example, in Western Kenya, GROOTS, a network of community-based organisations, designed a programme to reduce women’s HIV risk at the community level by protecting and enhancing women’s access to and ownership of land [37]. Other organisations choose to work with the criminal justice system, using legal empowerment to tackle violence against vulnerable populations, particularly sex workers and drug users, ensuring that incarcerated individuals have access to health services to which they are entitled [36, 40, 41, 47].

### Enabling factors and constraints

The empirical articles reviewed identified several important factors for success as well as constraints. Because the evidence is thin, we also draw on background articles (i.e. commentaries and empirical analyses of legal empowerment programs outside of health) and interviews to ground our analysis in the legal empowerment field more broadly. Table 4 describes enablers, and Table 5 describes constraints drawn from the empirical articles, background articles, and interviews. We present these in tables rather than text, as many of the factors are fairly intuitive (*i.e.* an unsurprising finding) and require little explanation.

**Table 4:** Enabling factors for legal empowerment programs

Factor	Explanation and caveats	Citation
Paralegals come from the communities they serve	• Builds trust with community • Ensures that the paralegal understands key community issues, fostering empathy and ability to go beyond formal methodologies and use local problem solving • More complicated in humanitarian contexts where ‘peers’ may not be familiar with local administrative procedures • In situations where they come from local elite; they may reinforce status quo power relations	([24, 35, 41, 42, 47, 48, 49, 50, 51]; interviews)
Legal empowerment programme personnel have relationships with organisations and individuals in the governmental and non-governmental sector	• Facilitates referrals to and from complementary services • Facilitates resolution of barriers to effective public sector health care and other service coverage • Protects program personnel from harassment in contexts with restricted civic space	([9, 24, 41, 47, 50]; interviews)
Legal empowerment activities are undertaken as part of a broader ecology (implemented by the legal empowerment organisation and/or others) of efforts to improve empowerment and health service delivery	• Legal empowerment activities are often undertaken in tandem with strategic litigation, legal aid, and political advocacy • Legal empowerment organisations create a network of legal empowerment advocates and providers, including for example, paralegals, volunteers, community groups • Political advocacy helps to create the conditions for sustainable impact • Legal empowerment can affect improvements in effective health service coverage, but can be strengthened by efforts to build health system capacity	([5, 9, 24, 25, 34, 35, 41, 43, 44, 52]; interviews)
Legal empowerment programme is able to respond to emergent community needs, and produces early successes	• Builds trust and relationships with the community • Mobilizational effects	([8, 42]; interviewees)
Use of customary or alternative dispute resolution	• In some cases, can be more trusted by community, more participatory, faster, and more impactful	[24, 35, 41]

**Table 5:** Constraints on legal empowerment programs

Factor	Explanation	Citation
Inconsistency in paralegal capacity	• Paralegals not always supported after training • Paralegals come from marginalised communities, and as such, may require significant training and support to learn about rights, entitlements, and the details of health policy • Paralegals may be overwhelmed with case-loads and lack the time and support to think about and address the upstream causes of the cases • Frequent turnover, especially among volunteer cadres, loss of institutional memory; may be more common among women, who may be focus of the programme	([9, 34, 40, 48, 49, 50, 51, 52]; interviews)
Lack of formalised role for paralegals	• In some countries, paralegals and LE organisations are seeking formal recognition for paralegals in national law, as well as accreditation processes • When roles are not recognised or ‘registered’ no customised training for them	([50, 52]; interviews)
Even with support, formal judicial or other processes can be inaccessible or infeasibly long	• Even when paralegals understand processes, they can be long; marginalised individuals may lack the time or may lose legal personhood over the course of case resolution or belief that the process will bear fruit	([24, 42]; interviews)
Customary law processes can reinforce social inequities	• In cases where those claiming rights are marginalised, the customary system may reproduce such inequities, especially when they are mediating between two parties, e.g. a poor woman who has experienced discriminatory treatment by a health provider	([49]; interviews)
Poor state capacity to respond	• Even where public officials are motivated, they may lack the resources, incentives, and/or expertise required to respond to complaints • Making demands on an ill-equipped bureaucracy can result in failure or even retaliation	([9, 24, 53]; interviews)
Unclear entitlements	• When rights and entitlements are not well enumerated (e.g. what drugs should be available at primary health care level?) then use of legal empowerment is impractical	([34, 44]; interviews)
Social hierarchies	• Pervasive discrimination and other norms can undercut individual and institutional responsiveness to complaints from groups/communities (e.g. drug users, ethnic minorities) • Paralegals from minoritised communities can face risks and stigma when approaching individuals with more power as well as state institutions	([35, 40, 45, 47]; interviews)
Donor priorities not aligned with community need	• Many programmes are donor driven and are siloed from broader state processes • Programme accountability is typically upwards to donors rather than to communities • Short term programmes aiming for long term change face challenges • Funder reluctance to support NGOs to take up politically sensitive issues	([49, 50, 54]; interviews)

Some of the strengths and weaknesses identified are two sides of the same coin, revealing some key trade-offs in programme design and implementation. For example, the use of customary structures may be more efficient, trusted, or feasible; these may also reinforce existing inequities. Similarly, having paralegals come from the communities – however that is defined—served by the programme appears preferrable, but this may have significant implications in terms of required technical support, as well as support for paralegals who experience significant backlash to their work.

## Discussion

This review of empirical articles on the use of legal empowerment in improving access to quality health services highlights the thinness of published evidence in this emerging area of work. The limited evidence is likely due in part to siloing between the fields of legal empowerment and public health. Few organisations work across and use both approaches. We attempt to bridge this divide by presenting our findings using both a public health (impacts on AAAQ), and a governance (strategic and programme approach) lens, distilling the challenges of implementation.

A few key insights emerged. Legal empowerment is generally focused on issues that communities themselves can assess, and often implemented in conjunction with an array of complementary approaches addressing communities as well as policy. Current programmes and research treat social hierarchies and health disparities as both targets of change and important contextual factors. It is unsurprising that such importance would be accorded to these issues. The limited body of research offers insight into how a strategy that pursues collectivisation and individual remedy can contribute to structural change, namely by facilitating individual access to justice and other services that relate to the social determinants of health, and by addressing governance determinants of health service delivery. Clear institutional channels and relevant information for individuals to access appropriate authorities both within and outside of the health sector can enable people to direct claims and seek resolution effectively [39]. Some programmes go beyond the health system to navigate the multiple institutions that exist at the local level, as these might be better placed to help solve problems than the health system in limited resources settings [34, 40].

In addition to ‘resolving’ problems and challenges, legal empowerment can entail building the capacity of service providers and other duty bearers on rights and entitlements enshrined in law and policy, as well as in participatory processes [40]. This has in some cases had the simultaneous effect of reducing outright abuse as well as improved relationships between communities and public officials [47]. It can be accomplished organically, such as health care providers learning how to solve common problems through observing paralegals. Paralegals themselves can also educate and support health care workers, such as through drafting letters to district officials in conjunction with health facility staff, or training duty bearers on the rights and entitlements of particular communities [8, 47].

Further, organisations with strong roots in the community, who are bound to represent their constituency have greater sustainability, legitimacy, and lasting power in the long run, as opposed to groups that are implementing short-term donor funded programs [39]. Donor funded programmes may prefer not to challenge state institutions and may “channel NGOs to moderate rather than radical goals” [36]. External legitimacy, in terms of relationships with state institutions also matter, as strengthened relations among community actors and the panoply of local level institutions can “shape the sustainability and transformative potential of the immediate improvements in service coverage and quality that result from resolving individual complaints” [9], pg. 11). And yet there may be trade-offs. Legal empowerment programmes that focus most on donor or government defined priorities may enjoy more legitimacy vis these actors, but less within their own community.

Existing documentation is primarily about identifying challenges and constraints to developing the legal empowerment approach, which could be a result of the fact that the legal empowerment field has largely been driven by lawyers rather than public health professionals. There is almost no literature specific to legal empowerment and health; the health systems perspective is largely absent from the literature. At the same time, as our interviewees pointed out, of the issues that legal empowerment can tackle, health is an area where there are generally common interests between rights holders and duty bearers; both parties want to improve population health. This is distinct from other areas, such as land disputes, where the outcome is often zero-sum. This may mean that the field of legal empowerment and health can evolve somewhat distinctly from legal empowerment more broadly, as issues such as cooperation and trust have been shown to be key to health systems effectiveness [18, 19, 55]. This type of learning has seemingly been limited in part because the field as a whole is dominated by individuals and organisations working starting from a legal paradigm, with less engagement from public health actors.

Another issue that the review raises is the need for capacity building on both the health and legal sides. On the legal side, both paralegals and those who are in a supporting or supervisory role for paralegals, need to develop skills to facilitate community-led agenda and priority setting. This includes seeing communities and groups as equal partners, valuing the knowledge and experience they bring, listening, and assenting to stepping back when pursuing formal processes does not ‘feel right.’ Similarly, on the health side, often health workers have limited knowledge of the health rights under the law—and increasing their awareness is frequently necessary as a part of legal empowerment programmes. Moreover, health professionals need to be capacitated to engage constructively with communities. Community Health Worker cadres are conceptually similar to paralegals – they liaise between communities and the health system, bringing information and services to communities and representing community concerns to the health system [56]. Deepening or systematizing their engagement in legal empowerment for health could help to ensure that efforts grounded in health system realities. Such an approach has the added benefit of communities becoming their allies in their advocacy for more resources and work conditions within the health bureaucracy.

The review also revealed some key gaps. First, was the lack of work on private sector health services, as a duty bearer or with regards to governments’ role in regulating such services. The quality of private sector health services and poor regulation are increasingly recognised as key in the health systems research literature [57, 58, 59]. Programmes promoting legal empowerment for health are usually based on government obligations,these obligations may not be invoked frequently in the context of private sector regulation, though they are almost always enshrined in domestic law. Lack of private sector engagement is likely due to political economic factors, namely the power of private sector actors who do not want to be subject to scrutiny or rights claims. The extent to which legal empowerment strategies might be used to spur government action in regulation or in demanding remedy and redress for people whose rights are violated by private sector health providers is a potential area of programme development.

Second, while empowerment is a common theme, there is little discussion or evidence regarding to what extent marginalised individuals participate in legal empowerment programmes, the extent to which they are empowered, and what defines empowerment. In the papers reviewed, empowerment was assumed and often equated with increased awareness and participation in processes of demanding redress. Whether power is actually rebalanced, both between communities and public officials (including health providers), and within communities between different groups is generally not addressed. Whether marginalised individuals can act on newly acquired entitlements knowledge, overcome gender norms, other hierarchies, and stigma to interact with customary or administrative systems, and actually benefit from any redress offered remains an important open question. Moreover, this raises programme design questions that are under-explored in the literature. For example, how can paralegals be trained and supported to accompany marginalised individuals through administrative processes, or to speak out in participatory fora?

Finally, there is also the danger of empowering people to confront authorities about rights violations without adequate understanding of the risks. Given that we know processes of empowerment require sustained long-term investment, and that externally supported legal empowerment programmes can be term limited projects, how do we understand the risks of such processes, particularly in contexts of restricted civic space, repression and legacies of fear and low expectations? Risk is emerging as a key consideration in the social accountability literature, where individual and collective demands for change can be met by retaliation from the very health providers on whom one depends [21]. Although some interviewees spoke about risk and fear and the interplay between empowerment and retaliation, this concern was largely absent from the empirical articles.

### Limitations

One of the limitations of the approach taken by this paper and related peer reviewed literature, is that these papers examine the fairly narrow question of the impact of legal empowerment and health programmes. These do not typically explore the question of what problems legal empowerment is best placed to address. In other words, the starting point of these papers generally follows the logic of programme monitoring and evaluation, rather than the more fundamental question of “what is the health systems problem we are trying to solve?” We reproduce this logic in our review, in part because few papers tackle these concerns. While there is increasing recognition of the pertinence of problem-driven approaches in development, we have adopted a programme implementation lens.

Second, as is the case for many domains, the peer-reviewed and the grey literature likely do not reflect the richness of programmes and efforts being undertaken, particularly those led by grassroots NGOs and other actors – such as governments – who are not funded by international donors and/or who do not have the time or see a benefit in publishing their experiences. The work reviewed here is dominated by donors working in HIV, potentially skewing the programmes and the literature.

In addition, many organisations do not label what they do as legal empowerment. Rather, given the number of approaches that are closely related to legal empowerment, organisations and funders could be grouping work under ‘social accountability’, human rights or social inclusion headings. While these distinctions are less meaningful in practice on the ground, they do mean that their work would not be found by our search terms, and the lessons this work offers would go unnoticed.

Also, given the potential political sensitivity of legal empowerment, it is quite likely that some papers may purposely not emphasise or disclose the more adversarial elements of their work, particularly in settings where this approach needs to be balanced with cooperation with the government at the national level. This is likely exacerbated by publication bias, which tends to favour success over failure, and cooperative over adversarial approaches. Relatedly, many of the papers reviewed provided very brief descriptions of legal empowerment programmes, making it difficult to distil lessons.

Moreover, as noted, some donors fund multiple programmes as part of a larger initiative, and together, these programmes meet our definition of legal empowerment. Because these programmes are often undertaken by different NGOs, it is quite likely that this multi-project approach is under-represented in our findings. Moreover, this collaboration could occur organically without donor intervention, such as through coalitions that aim to promote health rights and entitlements. These experiences are also likely under-represented in the peer-reviewed and grey literature.

## Conclusions

Efforts to support the development of the field might start by supporting organisations that are working in this space to carry out accompanying documentation and research. As indicated earlier, many organisations are practising strategies that might be labelled legal empowerment, but they do not self-identify as such and therefore are not part of the evidence base of what we know from experience about the use of legal empowerment in the health field. Support for the identification, documentation and analysis of such experience seems to be a priority for building the field.

In the process of such documentation, some issues need to be examined in greater detail. In particular, we need better understandings of the ‘empowerment’ aspects of legal empowerment, of the risks and sustainability of the approach, and of its contributions to structural change. Greater grounded empirical evidence on these issues will enable practitioners and policy makers to make informed decisions about how to leverage the legal empowerment approach in health most effectively.

Overall though, we argue that foremost, it is important to place legal empowerment in the broader context of the problem one is trying to solve – in other words the ultimate objectives. In some contexts, paralegals could potentially relieve pressure on the state to ensure access to justice [54]. Critical legal empowerment offers the perspective that the law and/or the way it is enforced may in itself be a mechanism for marginalisation. While many programmes we read and heard about could potentially contribute to a more just health or justice system, the structural impacts beyond channels for dialogue, remedy, and trust at the local level were rarely explored in empirical research. The under theorisation and exploration of empowerment, risk, and equity inhibits our understanding of the relationship between short term local change, and broader structural change. The call for more explicit power analysis is not confined to health; in the broader LE field, there have been calls for planning and implementing legal empowerment programmes in the broader political economic context, and thinking through how legal empowerment can shift that context [5].

Finally, there needs to be greater recognition that legal empowerment is not the silver bullet that can magically improve health services—it is better suited to certain kinds of problems within health systems and not others. Asking questions such as: Are there particular types of health service issues that LE is better able to address? Are particular types of marginalisation or lack of access better served by specific LE approaches (e.g. GBV)? Can a right to health framing benefit legal empowerment efforts in other sectors, such as related to extractive industries? will be important in advancing the field to tailor legal empowerment approaches and better target health challenges.

## Supplementary Information

**Additional file 1:** **Supplemental Table 1.** Articles included in the review.

**Additional file 2:** **Annex 2.** Articles reviewed.

## Data Availability

All data generated or analysed during this study are included in this published article.

## Abbreviations

LMIC: Low and Middle Income Country
UHC: Universal Health Coverage
AAAQ: Available, Accessible, Acceptable, Quality
CHW: Community Health Worker
CSW: Commercial Sex Worker
LE: Legal Empowerment

## Footnotes

1 The field of legal empowerment is somewhat distinct in high income countries, where it has evolved as an element of legal aid. In addition, the process that launched the field—the Commission on Legal Empowerment and the Poor – focused largely on LMICs. Though we do not want to reproduce a dichotomy between high income and LMICs, we recognise that the field itself largely mirrors this dichotomy.

2 In addition to comprehensively reviewing the literature on legal empowerment and health and low- and middle-income countries, the funder (IDRC) was particularly interested in West Africa and the Middle East and North Africa. For this reason, we included French and Arabic searches, in addition to English. As noted, we identified no resources in French, and a few background papers in Arabic. The general lessons drawn from these papers were consistent with those in English language materials. For this reason, we do not think that the additional languages biased our findings towards West Africa or the Middle East. Rather the inclusion of the additional languages strengthens our findings about the paucity of research.
