# Supplementary material for: The use of legal empowerment to improve access to quality health services: a scoping review
Source: Int J Equity Health. 2022 Sep 16;21:136. doi: 10.1186/s12939-022-01731-3 (PMC9482253; doi:10.1186/s12939-022-01731-3)
Supplement: Supplementary file 1 — Additional file 1: Supplemental Table 1. Articles included in the review. [file 12939_2022_1731_MOESM1_ESM.docx]

Supplemental Table 1: Articles included in the review

| **Article title** | **Authors** | **Country** | **Type of article** |
| --- | --- | --- | --- |
| Expanding the role of paralegals: supporting realization of the right to health for vulnerable communities | (Wirya, Larasati, Gruskin, & Ferguson) | Indonesia | research article |
| Legal empowerment approaches in the context of COVID-19 | (Dhital & Walton) | Argentina, India, US | Commentary with three brief program summaries, focused on NGOs reactions to COVID-19. Only India program fits our definition of legal empowerment. |
| Access to justice: evaluating law, health and human rights programmes in Kenya | (Gruskin, Safreed-Harmon, Ezer, Gathumbi, Cohen & Kameri-Mbote) | Kenya | research article on 3 programs |
| Assessing Legal Advocacy to Advance Roma Health in Macedonia, Romania, and Serbia | (Abdikeeva, Ezer & Covaci) | North Macedonia, Romania and Serbia | program summary |
| Namati: Innovations in Legal Disempowerment for Health? A Sierra Leonean Case Study | (Achilihu) | Sierra Leone | research paper; Master's Thesis |
| What community-level strategies are needed to secure women’s property rights in Western Kenya? Laying the groundwork for a future structural HIV prevention intervention | (Dworkin, Lub, Grabec, Kwenad, Mwaura-Muirue & Bukusid) | Kenya | research |
| Can sex workers regulate police? Learning from an HIV prevention project for sex workers in southern India | (Biradavolu, Burris, George, Jena, & Blankenship) | India | research |
| Examining the Effectiveness of Legal Empowerment as a Pathway out of Poverty: A Case Study of BRAC | (Kolisetty) | Bangladesh | program summary |
| Legal Empowerment Within the Primary  Healthcare Delivery | Network for Movement for Democracy and Human Rights | Sierra Leone | program summary |
| Transforming Policy into Justice-The Role of Health Advocates in Mozambique | (Feinglass, Gomes, and Maru) | Mozambique | program summary |
| Breaking the links: Legal and paralegal assistance to reduce health risks of police and pre-trial detention of sex workers and people who use drugs | (Wolfe, Cohen, Doyle, and Margolin) | Ukraine, Kenya, and Indonesia | commentary with three short cases |
| A Right-Based Approach to Lawyering: Legal Empowerment as an Alternative to Legal Aid in Post-Disaster Haiti | (Jagannath, Phillips and Shah) | Haiti | commentary with two cases |
| Do more empowered citizens make more accountable states? Power and legitimacy in legal empowerment initiatives in Kenya and South Africa | (Feruglio) | only Kenya meets our inclusion criteria | Research report |
|  |  |  |  |
| We all have the same right to have health services': a case study of Namati's legal empowerment program in Mozambique | (Schaaf, Falcao, Feinglass, Kitchell, Gomes & Freedman) | Mozambique | research |
|  |  |  |  |
|  |  |  |  |
|  |  |  |  |
|  |  |  |  |
|  |  |  |  |
|  |  |  |  |
|  |  |  |  |
|  |  |  |  |
| Legal Empowerment and Social Accountability: Complementary Strategies Toward Rights-based Development in Health? | (Joshi) | Guatemala, North Macedonia, Uganda, India | research |
|  |  |  |  |
|  |  |  |  |
|  |  |  |  |
|  |  |  |  |
|  |  |  |  |
|  |  |  |  |
|  |  |  |  |
|  |  |  |  |
|  |  |  |  |
|  |  |  |  |
|  |  |  |  |
|  |  |  |  |
|  |  |  |  |
|  |  |  |  |
|  |  |  |  |
|  |  |  |  |
|  |  |  |  |
|  |  |  |  |
|  |  |  |  |
