# Supplementary material for: The use of legal empowerment to improve access to quality health services: a scoping review
Source: Int J Equity Health. 2022 Sep 16;21:136. doi: 10.1186/s12939-022-01731-3 (PMC9482253; doi:10.1186/s12939-022-01731-3)
Supplement: Supplementary file 2 — Additional file 2: Annex 2. Articles reviewed. [file 12939_2022_1731_MOESM2_ESM.docx]

Annex 2: Articles reviewed

| **Article title** | **Authors** | **Country** | **Type of article** | **LE program description** |
| --- | --- | --- | --- | --- |
| Expanding the role of paralegals: supporting realization of the right to health for vulnerable communities | (Wirya, Larasati, Gruskin, & Ferguson) | Indonesia | Research article | Lembaga Bantuan Hukum Masyarakat (LBHM) is a non-governmental organisation that provides law and human rights training to marginalised communities, such as people who use drugs, people living with HIV (PLHIV), LGBTQI, sex workers, and other communities. Prior to the training, LBHM deploys a team of legal counsellors to assess the situation of the target communities. During this series of trainings, LBHM tries to identify potential community members to be further trained as paralegals. |
| Legal empowerment approaches in the context of COVID-19 | (Dhital & Walton) | Argentina, India, US | Commentary with three brief programme summaries, focused on NGOs reactions to COVID-19. Only India programme fits our definition of legal empowerment. | Founded in 2012 and based in New Delhi, Nazdeek combines grassroots legal education, community monitoring, legal remedies, research, and advocacy. The organisation is a collective of women activist-lawyers, researchers, and community paralegals. The project described took place in an informal settlement, where women paralegals were trained on rights-based frameworks to build their skills to document, monitor, and file complaints. Nazdeek has also partnered with community members to file strategic litigation and has offered advocacy support. They have addressed lack of a local health clinic and nutritional centres and community health workers, as well as inadequate delivery of water and sanitation. |
| Access to justice: evaluating law, health and human rights programmes in Kenya | (Gruskin, Safreed-Harmon, Ezer, Gathumbi, Cohen & Kameri-Mbote) | Kenya | Research article on 3 programmes | The 3 programmes evaluated are implemented by NGOs, and take a 'legal integration' approach. They incorporate legal aid, training and representation into existing health services to improve health outcomes and advance human rights. Activities include on-site legal service provision, client referrals to health and legal services, information campaigns focused on health and human rights, and human rights training for health providers and health care service users. |
| The Impact of Legal Advocacy Strategies to Advance Roma Health: The Case of Macedonia | 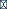 | North Macedonia | Programme summary | The programme was implemented by 3 different NGOs, meeting our definition of legal empowerment when defined. The programmes entailed training health paralegals; building capacity among Roma activists, particularly Roma women; the establishment of a health advising centre to promote human rights awareness among the community and advocacy for Roma rights before health authorities at different levels; and a legal department identifying strategic litigation opportunities and initiating cases. |
| Assessing Legal Advocacy to Advance Roma Health in Macedonia, Romania, and Serbia | (Abdikeeva, Ezer & Covaci) | North Macedonia, Romania and Serbia | Programme summary | Only Romania and Serbia are described here, as the North Macedonia programme is described in the previous record. In both cases, the programmes together meet our definition of legal empowerment. In Romania, a Bucharest-based NGO, Romani CRISS, carried out a project with two components: i) gather information about Roma rights violations in healthcare settings (with a special focus on segregation in maternity wards) as a basis for advocacy and ii) empower and build the capacity of two local Roma-centred human rights NGOs to address rights violations at the individual level. Another NGO, SASTIPEN, monitored discrimination against Roma in access to healthcare services, in part by modifying the procedures for resolving patients’ complaints before the local Colleges of Physicians. In Serbia, the NGO Bibija built the legal advocacy capacity of two NGOs focusing on Roma women, provided information to the Roma community about legal mechanisms for rights protection, and facilitated dialogues to encourage state institutions to monitor, document and resolve cases of Roma rights violations in healthcare settings. Another NGOs, Law Scanner, increased awareness among health-care professionals and the public about patients’ rights, and sought to strengthen the protection of human rights in healthcare settings. |
| Namati: Innovations in Legal Disempowerment for Health? A Sierra Leonean Case Study | (Achilihu) | Sierra Leone | Research paper; Master's Thesis | In its Accountability of Essential Services programme, Namati collaborates with the World Bank and the Ministry of Health and Sanitation (MoHS) to improve the quality and accountability of health care services for Sierra Leoneans. Paralegals provide a platform for communities to air their grievances to the MoHS and its decentralised organs and educate facility management committees (FMCs) on their roles in advocating for accountability in the health sector. These FMCs act as liaisons between health facilities and communities to ensure that health facilities are fulfilling their obligations as specified in the national health policy, and document failures for case mediation and for advocacy. FMCs rely on conflict resolution techniques to see if both parties can agree in the event that grievances arise. In addition to case mediation, mobilisation and education of rights holders and duty bearers regarding health entitlements is a key component to the project. |
| What community-level strategies are needed to secure women’s property rights in Western Kenya? Laying the groundwork for a future structural HIV prevention intervention | (Dworkin, Lub, Grabec, Kwenad, Mwaura-Muirue & Bukusid) | Kenya | Research | The NGO GROOTS-Kenya developed the programme “Community Land and Property Watch Dog Model” to address the fact that women were being disinherited, contributing to their vulnerability to HIV, and ultimately, to the spread of HIV. The model entails Watch Dog Groups (WDGs) comprised of community health workers, traditional leaders, trained paralegals, and government stakeholders. WDGs provided education regarding property rights to individual community members, created funeral committees that intervened to prevent property grabbing and disinheritance, provided paralegal training to traditional leaders and community members who adjudicated cases of property rights violations, and referred property rights violations to the formal justice system when they were not resolved at the community level. |
| Can sex workers regulate police? Learning from an HIV prevention project for sex workers in southern India | (Biradavolu, Burris, George, Jena, & Blankenship) | India | Research | The NGO described in the paper implemented a community-led structural intervention for HIV prevention among female sex workers. In the context of ongoing violence by the police, the NGO added a Crisis Intervention Team (CIT) to their ongoing prevention efforts. The CIT had a three-pronged approach: collective action and legal literacy support among sex workers so that they could confront improper police action; a rapid response process to mobilise individuals with power, including media, senior police officials, and political officials, to influence the police; and support to sex workers to file court cases if they were arrested, fined, or asked to pay a bribe. The programme was developed to accommodate non-literate sex workers; Community-based organisations were trained to document the sex workers' negative interactions with the police. |
| Examining the Effectiveness of Legal Empowerment as a Pathway out of Poverty: A Case Study of BRAC | (Kolisetty) | Bangladesh | Programme summary | BRAC’s Human Rights and Legal Aid Services programme is scaled up; it operates 517 legal aid clinics in 61 of 64 districts in Bangladesh. The programme relies on more than 6,000 community-based outreach workers, known as shebikas, or "barefoot lawyers." These shebikas work in diverse areas, including education, health, microfinance, water and sanitation, disaster relief, and agriculture. The shebikas train communities on their rights and entitlements in constitutional law and religious law, and connect community members with legal advice, representation, and mediation services provided at BRAC-run legal aid clinics. The legal aid clinics provide support to clients to participate in community-based dispute processes, run their own alternative dispute resolution processes, provide legal advice, and provide legal representation in court. In addition, workshops for community leaders, such as religious leaders, teachers, journalists, and local development community members, seek to increase gender and human rights awareness among village leaders and to encourage collaboration with BRAC’s programmes. These community leaders then form a Human Rights Implementation Committee (HRIC) and commit to promoting human rights. |
| Legal Empowerment Within the Primary  Healthcare Delivery | Network for Movement for Democracy and Human Rights | Sierra Leone | Programme summary | The programme includes selection and training of paralegals, awareness raising on rights and entitlements among community members, community monitoring (using a scorecard) of the local health facility, and paralegals managing "cases" that arise from the monitoring. Some of these cases are collective action problems that require dialogue; others are state failures that require pursuing formal administrative processes to resolve. |
| Transforming Policy into Justice-The Role of Health Advocates in Mozambique | (Feinglass, Gomes, and Maru) | Mozambique | Programme summary | The Namati programme in Mozambique consists of trained community Health Advocates (paralegals) supported by the Namati programme team. The program utilises legal empowerment and social accountability strategies to increase community member awareness of health policies and rights, pursue solutions to specific breakdowns in health care delivery, and facilitating dialogue between communities and clinics. The Health Advocates pursue solutions to problems related to access, infrastructure, and provider performance through dialogue with the individuals concerned, engagement with health facility leadership, and, where necessary, engagement with district officials or the judiciary. Health Advocates also work with Village Health Committees to enhance their engagement in the right to health, including bringing "collective cases" that affect many individuals to the health system for resolution. |
| Breaking the links: Legal and paralegal assistance to reduce health risks of police and pre-trial detention of sex workers and people who use drugs | (Wolfe, Cohen, Doyle, and Margolin) | Ukraine, Kenya, and Indonesia | Commentary with three short cases | In Kenya, the programme consisted of raising community awareness through legal training, and ran training workshops where police and sex workers together learned relevant human rights and Kenyan law pertaining to sex work and due process. In Indonesia, individuals vulnerable to police harassment and detention, namely drug users and fishermen deemed illegal residents in Jakarta, are trained as paralegals to educate and support their peers about due process and other legal issues. In Ukraine, drug users and other HIV affected populations are trained as community paralegals in the minutiae of arrest procedures, so that they can use their knowledge as a negotiating tactic to persuade police to release a client rather than proceed with a trial that could easily result in a wrongful conviction. |
| A Right-Based Approach to Lawyering: Legal Empowerment as an Alternative to Legal Aid in Post-Disaster Haiti | (Jagannath, Phillips and Shah) | Haiti | Commentary with two cases | This programme was implemented by an NGO that consists primarily of lawyers. The activities describe that meet our inclusion criteria are services for women who have experienced gender-based violence. The lawyers provide legal empowerment as an alternative to legal aid by using organising and community engagement alongside legal representation. This strengthens the capacity of Haiti’s judicial system while opening up opportunities for community voice. To achieve this, the NGO employs "grassroots coordinators” to reinforce the media and governmental advocacy capacity of grassroots groups that work on the same issues for which clients seek legal assistance. |
| Do more empowered citizens make more accountable states? Power and legitimacy in legal empowerment initiatives in Kenya and South Africa | (Feruglio) | only Kenya meets our inclusion criteria | Research Report | The relevant programme from Kenya is a Bar Hostess Empowerment and Support Programme (BHESP) which aims to influence policy, facilitate the provision of quality health services, and facilitate human rights empowerment for sex workers, women who have sex with women, women using drugs and bar hostesses in Kenya. BHESP accomplishes this through health and legal awareness and counselling, legal representation in individual and collective cases of discrimination and abuse against sex workers, and awareness-raising services for health providers and police officers. Community level work is complemented with engagement in national level health policy discussions. |
|  |  |  |  |  |
| We all have the same right to have health services': a case study of Namati's legal empowerment program in Mozambique | (Anonymous al., 2020) | Mozambique | Research | The programme is being implemented by Namati. The programme strategy involves: (1) increasing community awareness of health policy, (2) strengthening community involvement in health governance, (3) pursuing solutions to specific breakdowns in health care delivery, and (4) using grassroots data to impact national policy and practice. Health Advocates (paralegals) undertake the first three activities through health entitlement information sessions in health facility waiting areas and schools, with VHCs, and with grassroots organisations; training and support Village Health Committees to undertake bi-annual health facility assessments; and working with VHC members to identify and address individual and collective patient grievances, or cases. Advocates work with the client or the VHC to shepherd these concerns through the administrative system. |
|  |  |  |  |  |
|  |  |  |  |  |
|  |  |  |  |  |
|  |  |  |  |  |
|  |  |  |  |  |
|  |  |  |  |  |
|  |  |  |  |  |
|  |  |  |  |  |
| Legal Empowerment and Social Accountability: Complementary Strategies Toward Rights-based Development in Health? | (Joshi) | Guatemala, North Macedonia, Uganda, India | Research | At the time the paper was written, in Guatemala, the Centro de Estudios para la Equidad y Gobernanza en los Sistemas de Salud (CEGSS) accomplished their work via a survey tool focused on the services that are most important to the indigenous communities that are a focus of their work, gathering narratives of cases where denial of health care had resulted in serious consequences, and bringing violations identified through both activities through the formal grievance and legal processes available. Community Health Defenders were trained to lead collective deliberation, prepare detailed documentation of the cases, and work with CEGSS to bring these cases to the four specialised authorities that deal with human rights violations. In Uganda, the Center for Health Human Rights and Development (CEHURD) uses community education, litigation and lobbying of parliamentarians to identify and address specific gaps in health rights fulfilment, as well as to monitor the implementation of policies intended to address those gaps. In North Macedonia, several NGOs provide legal aid, community education, and community monitoring - through NGO staff and paralegals - to Roma who are often excluded from the Health Insurance Fund and health service access. In India, Nazdeek addresses maternal and infant health issues among Adavasi (tribal) tea workers in Assam, who rely on tea plantations for all their needs, including healthcare. Nazdeek later expanded their work to include labour rights, based on the priorities of local groups and workers. Nazdeek partnered with a local organisation to train paralegals to document failures to fulfil maternal healthcare entitlements in the plantations using SMS. They also implemented awareness raising, collective deliberation, and community mobilisation. The crowd-sourcing approach to documenting cases enabled broader data collection for advocacy as well as follow-up of particularly serious cases of rights violations. |
|  |  |  |  |  |
|  |  |  |  |  |
|  |  |  |  |  |
|  |  |  |  |  |
|  |  |  |  |  |
|  |  |  |  |  |
|  |  |  |  |  |
|  |  |  |  |  |
|  |  |  |  |  |
|  |  |  |  |  |
|  |  |  |  |  |
|  |  |  |  |  |
|  |  |  |  |  |
|  |  |  |  |  |
|  |  |  |  |  |
|  |  |  |  |  |
|  |  |  |  |  |
|  |  |  |  |  |
|  |  |  |  |  |
